# Achieving Long Cycle Life of Zn-Ion Batteries through Three-Dimensional Copper Foam

**DOI:** 10.1021/acsami.4c01028

**Published:** 2024-04-25

**Authors:** Taşkın Çamurcu, Erhan Demirbaş, Mehmet Nurullah Ateş

**Affiliations:** † Gebze Technical University, Department of Chemistry, 41400 Gebze, Kocaeli, Türkiye; ‡ Bogazici University, Department of Chemistry, Bebek 34342, Istanbul, Türkiye; § TÜBİTAK Rail Transport Technologies Institute, Energy Storage Division, TÜBİTAK, Gebze Campus, 41470 Gebze, Kocaeli, Türkiye

**Keywords:** aqueous batteries, zinc-ion batteries, 3D copper foam, thin foam, current collectors

## Abstract

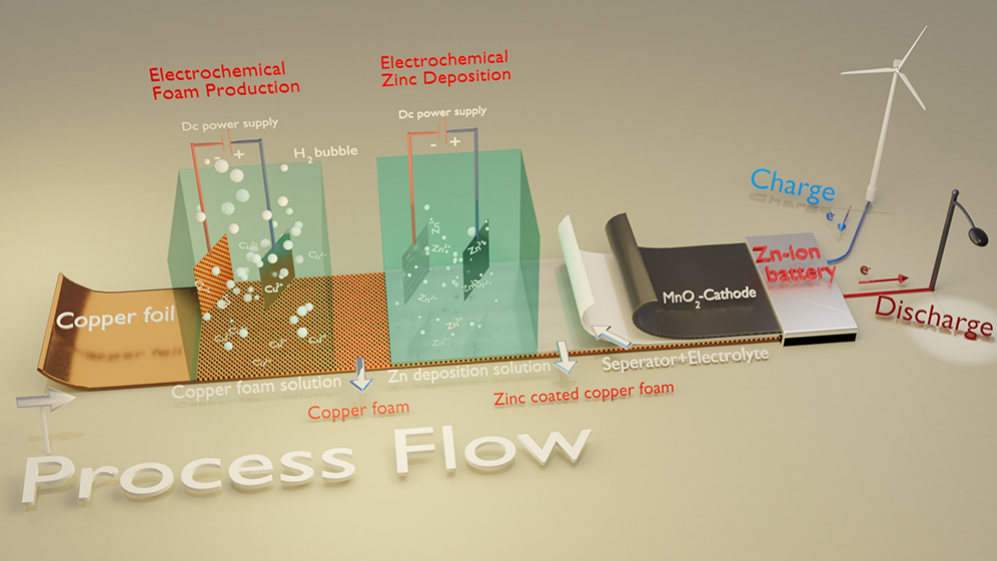

Metallic zinc anodes in aqueous zinc-ion batteries (ZIBs) suffer from dendritic growth, low Coulombic efficiency, and high polarization during cycling. To mitigate these challenges, current collectors based on three-dimensional (3D) commercial copper foam (CCuF) are generally preferred. However, their utilization is constrained by their thickness, low electroactive surface area, and increased manufacturing expenses. In this study, the synthesis of cost-effective current collectors with exceptionally large surface areas designed for ZIBs that can be cycled hundreds of times is reported. A zinc-coated CuF anode (Zn/CuF) was prepared with a 3D porous CuF current collector produced by the dynamic hydrogen bubble template (DHBT) method. Electrochemically generated copper foam could be obtained within seconds while offering a thickness as low as 30–40 μm (CuF5 achieved a thickness of ∼38 μm in 5 s) via the DHBT method. The excellent electrical conductivity and open pore structure of the 3D porous copper scaffold ensured the uniform deposition/stripping of Zn during cycling. During the 500 h Zn deposition/stripping process, the as-synthesized CuF5 current collector offered fast electrochemical kinetics and low polarization as well as a relatively high average Coulombic efficiency of 99% (at a current density of 5 mA cm^–2^ and a capacity of 1 mAh cm^–2^). Furthermore, the symmetric cell exhibited low voltage polarization and a stable voltage profile for 1000 h at a current density of 0.1 mA cm^–2^. In addition, full cells containing the Zn/CuF anode coupled with an as-synthesized α-MnO_2_ nanoneedle cathode in aqueous electrolyte were also prepared. Capacities of 266 mAh g^–1^ at 0.1 A g^–1^ and 94 mAh g^–1^ at 2 A g^–1^ were achieved after 200 charge/discharge cycles with a stable Coulombic efficiency value close to 99.9%.

## Introduction

In order to integrate renewable energy with electrochemical energy storage, innovative, secure, and affordable large-scale energy storage technologies must be developed.^1^ For large-scale energy storage system applications, lead acid batteries, redox-flow batteries, and lithium-ion batteries (LIBs) are now the most common electrochemical energy storage technologies.^2,3^ The cost, security, and sustainability of energy storage systems are crucial for large-scale applications.^4,5^ The large-scale implementation of LIBs is constrained by a shortage of lithium supplies, volatile organic electrolytes, and security concerns.^6^ Although lead acid batteries are safe and reasonably priced, their short cycle life and low power density limit their application in large-scale storage systems.^7^ Zinc-ion batteries (ZIBs), which are considered alternative energy storage systems to satisfy low-cost and high-safety standards, are therefore receiving gradually increased attention due to their nontoxic aqueous electrolytes and generally abundant electrode materials.^8^

Metallic zinc is used as an anode material in many battery systems, including Zn//Ni, Zn//Ag, Zn//air, Zn//MnO_*x*_, and Zn//VO_*x*_, due to its unique properties such as its high theoretical capacity (819 mAh g^–1^, 5881 mAh mL^–1^), low electrochemical potential (0.762 V vs SHE (standard hydrogen electrode) in neutral electrolyte), and environmental friendliness.^9^ In recent years, several attempts have been made to create high-performance ZIBs. However, a number of problems still remain to be solved for the future deployment of ZIBs, particularly regarding the “hostless” Zn metal anode, which is responsible for the inadequate cycle stability and low Coulombic efficiency (CE) of ZIBs.^10^ The uncontrolled development of Zn metal dendrites during the Zn striping/plating process frequently results in short circuits, which reduces cycle life.^11^ Numerous studies have been carried out to solve the aforementioned problems. As an example of such studies, Yang et al. demonstrated that the pH change occurring at the interface significantly influenced dendrite formation. They synthesized a N-modified graphdiyne interface (NGI) to stabilize the pH, resulting in a 116-fold increase in the lifetime of the symmetric cell without any dendritic growth. This study highlights the importance of interface pH and interface engineering.^12^ As another example, Liu et al. designed ion-conducting channels by creating graphdiyne (GDY) nanowalls on the surface of a zinc electrode. It was claimed that these nanowalls provided a large surface area on the zinc surface, ensuring the homogeneous distribution of the current density and thus inhibiting zinc dendritic growth. Additionally, it was claimed that they achieved an 82% capacity retention and excellent cycle stability after 5000 cycles at a current density of 1 A g^–1^ in full-cell studies. This study serves also as a good example of surface engineering.^13^ In addition to these studies, surface protection of Zn metal, electrolyte design, optimization of substrate structure, and studies of new separators have been frequently studied topics.^14^

Among the many methods that exist, the idea of using a three-dimensional (3D) current collector stands out. Since 3D current collectors have a large specific surface area, they can reduce the current density; thus, they ensure the electric field is homogeneously distributed.^15−17^ This can inhibit the growth of dendrites during cycling.^14^ 3D current collectors can also deposit more zinc metal without changing the overall thickness compared to two-dimensional (2D) planar materials, mitigating the effect of volume change on the battery.^18,19^ For example, Shi et al. compared different commercial current collectors in a ZIB battery system and reported that Cu foam performed better.^20^ Although commercial 3D current collectors have solid structures and large pores, they are heavy, and the active surface per unit area is limited.^21^ Most of the current collectors reported in previous studies are successful carriers for zinc deposition, and the major difference between them is their intrinsic zinc nucleation overpotentials (ZNOs). A lower ZNO represents a lower energy barrier and a more uniform deposition behavior, and it enhances reversible deposition/stripping behavior and minimizes dendritic Zn growth.^22,23^ Based on this, as an alternative to commercial 3D structures, Kang et al. reported the fabrication of 3D porous copper scaffolds by the chemical etching of copper foil. The 3D porous copper scaffolds they produced were found to serve Zn deposition/stripping for 350 h.^16^ To effectively improve the irreversible plating/stripping behavior of the Zn metal anode, selecting a suitable current collector with a low ZNO and highly reversible behavior is the key point for success.^24,25^

The aim of this work is to synthesize inexpensive, scalable, thin, and large surface area copper current collectors with properties such as a low ZNO value, high cycling efficiency, and stability. The dynamic hydrogen bubble template (DHBT) method is used to produce 3D porous structures based on electrochemical deposition using the hydrogen bubble as a template,^26^ and it has been used in numerous electrochemical applications such as batteries, capacitors, wastewater treatment, sensors, and catalysts in the literature.^27^ We synthesized a 3D porous copper current collector by using the DHBT method and used it as a current collector for Zn-ion batteries. Zn metal was electrochemically coated on the 3D porous Cu foam (CuF) produced by DHBT to produce an anode electrode. As a result, although the foam is super thin with a thickness of about 38 μm, it is composed of dendritic copper, providing an extra-large active surface area, and the first plating showed a low nucleation overpotential. Moreover, asymmetric deposition/stripping cycling (at current densities of 1, 2, and 5 mA cm^–2^ and an areal capacity of 1 mAh cm^–2^) provided a low voltage hysteresis and a stable Coulombic efficiency for over 500 cycles. The symmetric cell constructed with the CuF5 foam exhibited high stable cyclability at a current density of 0.1 mA cm^–2^ with a limited capacity of 0.1 mAh cm^–2^ for over 1000 h. While the Zn/CuF5//α-MnO_2_ full cell built with the CuF5 anode and α-MnO_2_ cathode produced a capacity of 266 mAh g^–1^ at relatively low rates, a capacity of 90 mAh g^–1^ was obtained at 2 A g^–1^ even after 200 deep charge/discharge cycles.

## Experimental Section

### Materials Preparation

#### Synthesis of 3D Porous Cu Foam

**Figure 1:**
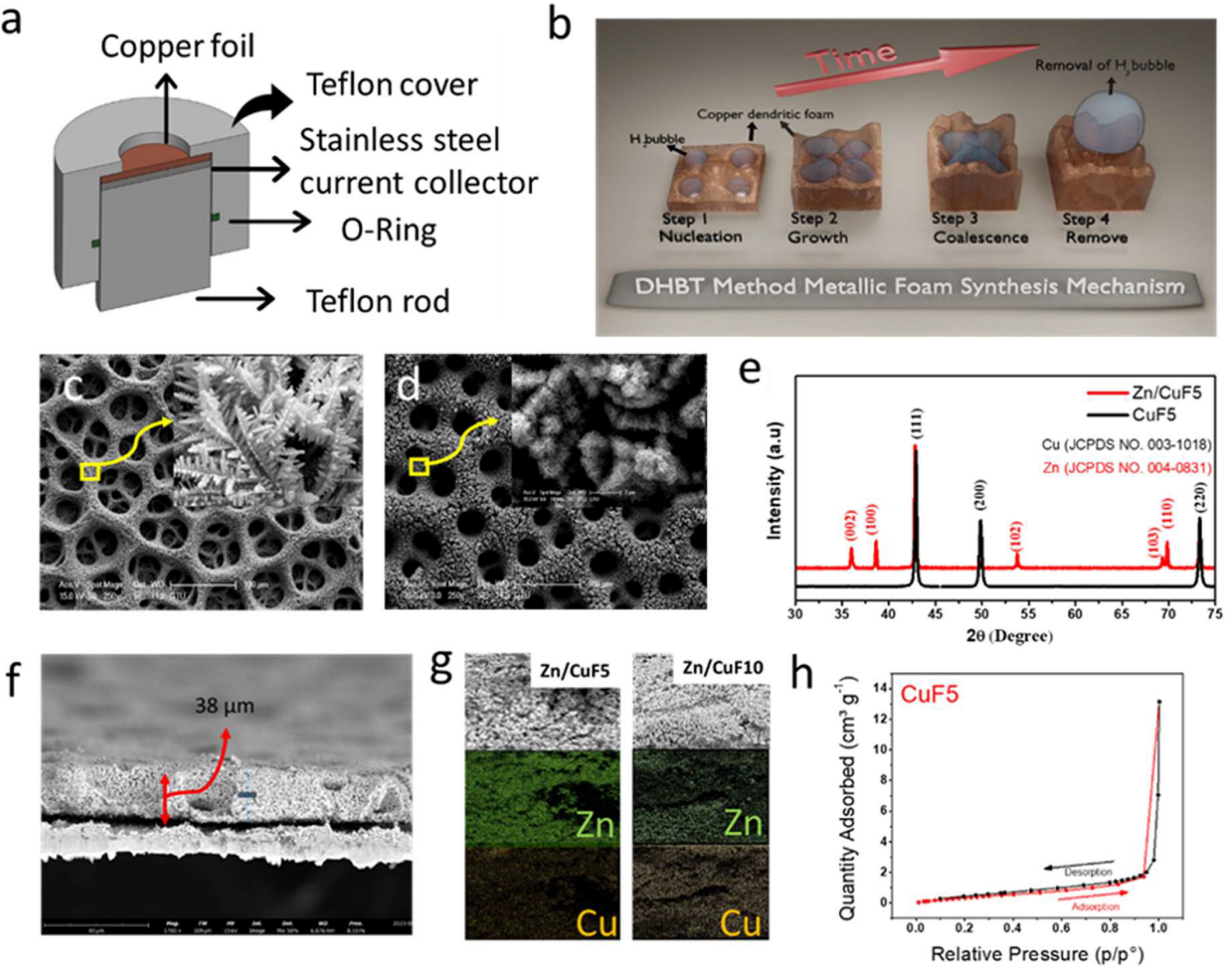
(a) 3D design of the custom-made electrode used for the synthesis of Cu foam. (b) Schematic illustration of the synthesis of porous copper foam (CuF). (c) SEM image of CuF5 (inner image: of nanodendrites forming the walls). (d) SEM image after electrochemical deposition of Zn on CuF5 (Zn/CuF5). (e) XRD patterns of CuF5 and Zn/CuF5. (f) SEM image of CuF5 cross-sectional thickness measurement. (g) Cross-sectional SEM and EDX mapping images of Zn/CuF5 and Zn/CuF10 (for Zn and Cu). (h) Brunauer–Emmett–Teller (BET) surface area data: nitrogen adsorption–desorption isotherms.

3D foam was produced on copper foil (Sigma-Aldrich) using a DC power source (Rigol DP832A) in a two-electrode system where an auxiliary electrode platinum plate (1 cm^2^) was used as a counter electrode, in accordance with the literature.^26^ Briefly, the Cu foil was first cleaned from the residual oil and oxide layer with a 10% HCl/ethanol/water mixture. Afterward, it was placed in a special electrode holder (Figure 1a illustrates a 3D image; for further details, see Figure S1) and immersed in an aqueous solution containing a mixture of 0.2 M copper(II) sulfate pentahydrate (CuSO_4_·5H_2_O), 1.5 M sulfuric acid (H_2_SO_4_, 95.0–98.0%), and 0.01 M sodium chloride (NaCl). To prepare the 3D foam, a constant current density of 2 A cm^–2^ was applied to the cell for various times (5, 10, and 20 s). The prepared 3D foam was washed 3 times with a water/ethanol mixture and dried in a vacuum oven (80 °C). The synthesized electrolyte was carried out at room temperature without stirring.

#### Synthesis of Zn/3D Porous Copper Foam Electrodes

Zn was electrochemically synthesized using a three-electrode system on all current collectors. In brief, 7.5 g of zinc sulfate heptahydrate (ZnSO_4_·7H_2_O), 7.5 g of sodium sulfate (Na_2_SO_4_), 0.72 g of sodium dodecyl sulfate (C_12_H_25_SO_4_Na), and 0.9 g of boric acid (H_3_BO_3_) were dissolved in 60 mL of deionized water.^27^ A current density of 40 mA cm^–2^ was applied to the electrodes in the prepared solution for 600 s. Hg/HgCl was used as the reference electrode, and a platinum plate electrode was used as the counter electrode.

#### Preparation of α-MnO_2_ Nanorod Powder

The synthesis of the α-MnO_2_ nanorod powder was completed by a hydrothermal method. In short, 3.042 g of manganese sulfate monohydrate (MnSO_4_·H_2_O, Sigma-Aldrich) was mixed with 40 mL of deionized water and magnetically stirred until a homogeneous solution was formed. Then, a 40 mL homogeneous aqueous solution of 0.474 g of potassium permanganate (KMnO_4_, Sigma-Aldrich) was prepared and added dropwise to the initial solution. The mixture was stirred with a magnetic stirrer for 30 min and transferred to a 100 mL Teflon-coated stainless-steel autoclave. It was then heated to 140 °C in a programmable oven at 5 °C min^–1^ and left for 12 h. Finally, the resulting suspension was centrifuged several times and washed with a mixture of water and alcohol. The final products were dried overnight at 80 °C in a vacuum oven.

### Characterizations

The microstructure, cross section, thickness, pore diameter, particle size, and surface morphology of the materials and electrodes were investigated by scanning electron microscopy (SEM, Thermo Fisher Phenom ParticleX). X-ray diffraction (XRD) data of the crystal structures of the current collectors were obtained by a Rigaku SmartLab instrument using a Cu Kα radiation source in the range of 2θ = 10–75°. The Brunauer–Emmett–Teller (BET) surface area and average pore diameter were determined using a Micromeritics 3Flex instrument.

### Assembly of Asymmetric Cells

Asymmetric cells were assembled by combining different metal current collectors with a zinc foil electrode. The working electrode consisted of various current collectors, while Zn foil was utilized as both the counter and reference electrodes. A 2 M ZnSO_4_ aqueous solution was used as the electrolyte. The separator used in this setup was a 15 mm glass microfiber membrane (Whatman GF/D). CR2032-type cell lines were prepared. A Neware BTS-4000 battery test system was used to evaluate the Coulombic efficiency (CE), cycle life, voltage hysteresis, and nucleation overpotential of the asymmetric cells. The tests were conducted at current densities of 1, 2, and 5 mA cm^–2^ and limited to a discharge capacity of 1 mAh cm^–2^. Zinc stripping was performed until a 0.5 V cutoff voltage was reached during charging.

### Assembly of Symmetric Cells

To evaluate the cycling stability, Zn/CuF5 or Zn/commercial Cu foam (CCuF) couples were utilized to make a decent comparison between our foam and commercial foam. The electrolyte was a 2 M ZnSO_4_ aqueous solution, and the separator used was a 15 mm glass microfiber membrane (Whatman GF/D). The cell was operated at a constant areal capacity of 0.1 mAh cm^–2^ and a constant current density of 0.1 mA cm^–2^ by using CR2032-type coin cells on a Neware BTS-4000 battery tester.

### Assembly of Full Cells

Zn/CuF5 and Zn/commercial Cu foam (CCuF) anode electrodes were separately assembled against a α-MnO_2_-containing cathode electrode. A 40 μL aqueous solution containing 2 M ZnSO_4_ and 0.5 M MnSO_4_ was used as the electrolyte, and the separator used was a 15 mm glass microfiber membrane. The cells were sealed in air and left for 10 h before electrochemical tests were performed. CR2032-type coin cells were packaged.

### Electrochemical Measurements

To determine the zinc plating/stripping behavior of symmetric cells and full cells containing the 3D porous copper electrode and different current collector electrodes, galvanostatic charge/discharge behaviors were measured with a Neware BTS-4000 battery tester. Full-cell cyclic voltammetry (CV) measurements were performed at a scanning rate of 0.1 mV s^–1^ between 0.8 and 1.8 V, and electrochemical impedance spectroscopy (EIS) measurements were performed with an amplitude of 10 mV from 100 kHz to 0.1 Hz. CV and EIS measurements were conducted with an Autolab PGSTAT204 electrochemical station. All studies were carried out at room temperature.

## Results and Discussion

### Structural Characterizations

3D porous copper foam (3D CuF) was successfully prepared electrochemically with the DHBT method on a planar copper foil, as schematically shown in Figure 1b. A 3D sketch showing the structure of the Teflon-made deposition apparatus was developed specifically for the synthesis of 3D CuF (Figure 1a), and photographs of the electrode are shown in the Supporting Information. Figure 1c shows an SEM image of a CuF sample with a 3D hierarchical structure, where we electrochemically deposited porous Cu for 5 s at a cathodic current density of 3 A cm^–2^ (which is called CuF5 hereafter). To examine the morphological change at different deposition times, the SEM images for 10 s (CuF10) and 20 s (CuF20) are provided in Figure S2. It is clear from these images that the longer the deposition time, the more the foam quality is distorted after 10 s. The SEM image in Figure 1c shows that the pore size of CuF5 varied from 10 to 50 μm and that the average foam wall thickness was around 20 μm. After the CuF5 electrode was coated with metallic zinc (Zn/CuF5), the pore structure was preserved, as shown in Figure 1d. However, it was measured that the pore diameter narrowed to approximately 20–30 μm, while the pore wall thickness increased to approximately 30–40 μm. This change in pore diameter was due to the thickening of the pore walls and Zn deposition, ultimately decreasing the pore size. The phase compositions of the CuF5 current collector and Zn/CuF5 electrode were investigated by XRD, and the patterns are given in Figure 1e. It is clearly seen from the XRD patterns that no oxide layer was formed during the electrochemical synthesis of the CuF5 current collector and after the deposition of Zn on CuF5. It was undesirable for an oxide layer to form on the surface due to its poor electrical conductivity.^28^ To measure the deposition layer thickness, CuF5 was cut laterally for a cross-sectional SEM image (Figure 1f), and it was found to have a thickness of approximately 38 μm. There were significant changes in the pore diameters, pore wall thicknesses, and foam thicknesses of the 3D foams. The SEM images shown in Figure S2 show that (a) CuF10 and (b) CuF20 were formed by increasing the deposition time. For example, based on the thickness measurements from the cross-sectional SEM image of the CuF10 electrode in Figure S2c, the thickness of the foam was approximately 60 μm (compared to CuF5, thickness of 38 μm). Thus, the thickness increased proportionally as the deposition time increased. Additionally, increasing the deposition time caused distortions on the foam surface and decreased homogeneity (see the SEM image of CuF20 in Figure S2b). In order to use the CuF5 current collector electrode as an anode in an aqueous Zn-ion battery system, an anode electrode was produced by depositing zinc on the CuF5 current collector for 600 s at a current density of 40 mA cm^–2^ at room temperature. This anode electrode was called Zn/CuF5. An SEM image of Zn/CuF5 is shown in Figure 1d. From this figure, it is clear that the Zn deposition on the CuF5 current collector was homogeneous and there were still pore openings. Inspecting this image closer, namely the inset image in this figure, it can be understood that there were microsized holes even after Zn deposition. It is known that an open pore structure contributes to the homogeneous distribution of the electrolyte and current distribution on the electrode surface.^29^ However, in the commercial copper foam (CCuF, see the SEM images in Figure S3a,b), where Zn was deposited under the same conditions, a homogeneous coating did not take place, and local accumulation occurred due to the low electroactive surface area (Figure S3b). Several studies in the literature have utilized commercial copper foam as a current collector in zinc-ion batteries.^20,30^ In these studies, half-cell tests revealed a low cycling stability and high nucleation potential. This is primarily due to the low surface area and high thickness of the copper foam (reported as 1.5 mm). In contrast, the porous copper foam presented in this study offers better electrochemical performance in half-cell and full-cell tests with much better cycling stability and low nucleation potential. This enhancement is achieved due to the higher electroactive surface area compared to that found in previous studies. A schematic of the growth mechanism for Zn in commercial foam compared to that in the foam developed in this study is depicted in Figure S3c. In addition, increasing the coating thickness may mean a larger surface area, but it was seen that the effective utilization rate of the foam decreased with the coating thickness. Figure 1g shows the cross-sectional SEM images, along with the energy dispersive X-ray spectroscopy (EDX) elemental mapping images, of the Zn/CuF5 and Zn/CuF10 anode electrodes. From these image and EDX mappings, it can be understood that the zinc density of the Zn/CuF5 anode electrode was more dominant than the copper density, while the zinc and copper densities were similar in the Zn/CuF10 anode electrode, thus verifying the higher amount of Cu in the pore structure. Figure S4 also reveals the EDX spectra, along with the elemental percentages, of the Zn-coated CuF5 and CuF10 electrodes. From this figure, it could be seen that the Cu10 current collector had about 54% atomic Zn, while the Cu5 current collector had about 87% atomic Zn. It was thought that this could be due to the mass transport limitations of Zn ions in the electrolyte, which were exacerbated across the *z*-direction of the foam.^31^ Furthermore, BET surface area analysis of the 3D CuF and CCuF samples was performed to determine physical properties such as surface area and porosity (Figure 1h for CuF5 and Figure S5 for CCuF), and the surface areas were calculated to be 5.68 and 1.78 m^2^ g^–1^, respectively.

### Electrochemical Performances of Asymmetric Cells

**Figure 2:**
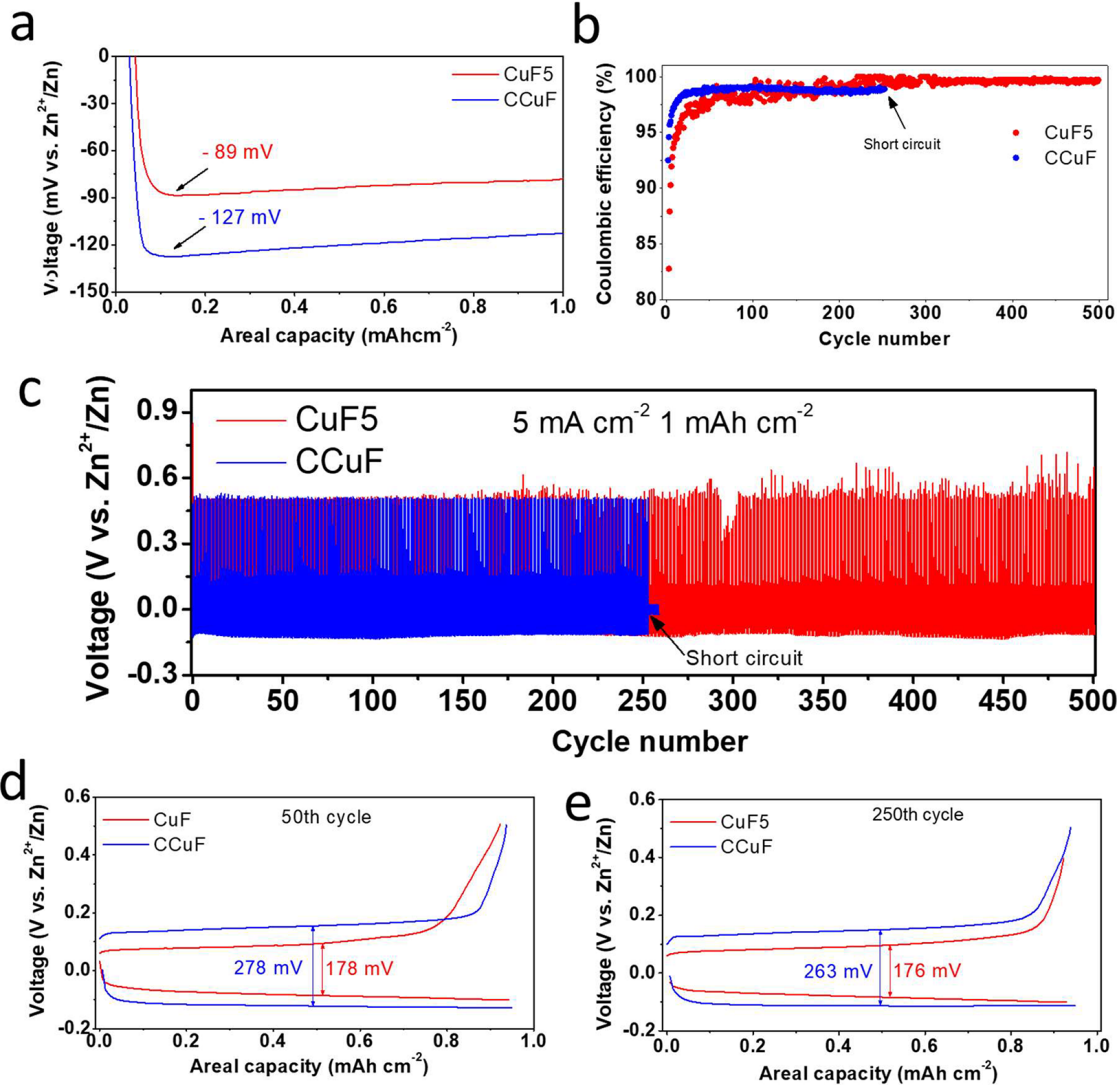
CuF5 and CCuF current collectors in asymmetric cells (against Zn electrode) at a current density of 5 mA cm^–2^ limited to a 1 mAh cm^–2^ areal capacity. (a) Zn deposition nucleation overpotentials, (b) corresponding Coulombic efficiencies during long-term cycling, (c) galvanostatic plating/stripping curves, (d) 50th galvanostatic plating/stripping voltage hysteresis, and (e) 250th galvanostatic plating/stripping voltage hysteresis.

In general, the electrochemical deposition of zinc on the anode surface is realized in several successive steps. In the first step, Zn^2+^ adsorption and electron transfer to the anode surface require activation energy to overcome the energy barrier. This is followed by the nucleation and growth steps, which play a critical role and directly affect the deposition quality. The galvanostatic voltage profile consists of two basic stages. First, there is a nucleation overpotential (nucleation overpotential between the peak and the trough), which is the point at where the voltage drops after the first few seconds of Zn deposition, followed by a plateau (plateau potential), which represents the process of film growth.^32^ In the initial nucleation phase at the anode surface, the energy barrier has to be overcome, and therefore, the nucleation overpotential is usually higher than the plateau potential. Both the nucleation overpotential and the plateau potential could be easily recognized in the galvanostatic profile. Zn//Cu asymmetric cells were assembled to investigate the nucleation and plateau potentials of CuF5 and CCuF. Figure 2a shows the initial galvanostatic discharge voltage profiles of the asymmetric cells with the CuF5 and CCuF current collectors limited to an areal capacity of 1 mAh cm^–2^ at a current density of 5 mA cm^–2^. In the literature, one of the common ways to establish the nucleation overpotential is the potential difference with respect to the 0 V axis.^20^ Accordingly, the nucleation overpotential was measured to be 89 mV for the CuF5 current collector and 127 mV for the CCuF current collector (Figure 2a). A difference of approximately 38 mV was observed between the two electrodes, and this difference did not change much during the plateau potential seen during the film growth. The nucleating overpotential difference measurements were repeated several times, and identical potential differences were observed, confirming the repeatability of the data.

CE measurements were performed to evaluate the reversibility of the CuF5 and CCuF current collectors in their half cells. CE represents the ratio of the stripping capacity to the deposition capacity of Zn ions. Figure 2b displays a comparison of the CEs for the CuF5 and CCuF current collectors with a limited capacity of 1 mAh cm^–2^ at a current density of 5 mA cm^–2^. The CuF5 anode exhibited a longer cycle life and higher efficiency than similar studies conducted in the literature,^33^ exhibiting a stable curve with an average CE of around 99% for 500 cycles, thus indicating its favorable Zn plating/stripping efficiency. After around 250 cycles, the cell containing CCuF experienced a short circuit, and the cell stopped working, which is likely due to dendritic growth of metallic Zn.^30^ The galvanostatic cycling data of these two half cells are shown in Figure 2c. In addition, an asymmetric cell with the CuF10 current collector was tested at a current density of 5 mA cm^–2^ (areal capacity limited to 1 mAh cm^–2^), and it was stable after approximately 100 cycles (CE of 93.61% for the first 100 cycles) (Figure S8). It was thought that this situation increased in direct proportion to the growth of the surface area.^20^ This may be because in the initial Zn plating/stripping process, the formation of the solid electrolyte interphase (SEI) film was accompanied by the consumption of some zinc ions. In subsequent cycles, the SEI was well formed, and metallic Zn could be deposited more thoroughly on the surface, thus achieving a high and stable CE.^11^ In general, for Cu foams, it was thought that a certain cycle number was required to reach a stable CE due to the high surface area. We also compared the CEs of the CuF5 and CCuF current collectors at 1 and 2 mA cm^–2^ (with a capacity of 1 mAh cm^–2^) (Figure S9a,b). At current densities of 1 and 2 mA cm^–2^, the CuF5 electrode performed for 500 cycles and showed the best cycling performance with CEs of 98.78% (at 1 mA cm^–2^) and 98.95% (at 2 mA cm^–2^). In Figure 2d,e, the voltage hysteresis of the galvanostatic charge/discharge curves of the 50th and 250th cycles of the Zn//CuF5 and Zn//CCuF asymmetric half cells is compared. In Figure 2d, the voltage hysteresis measured after the 50th cycle is 178 mV for the Zn//CuF5 cell and 278 mV for the Zn//CCuF cell. Similarly, after the 250th cycle (in Figure 2e), the voltage hysteresis of the CuF5-containing cell outperformed that of the CCuF-containing cell. Based on these results, we concluded that the CE of the CuF5-containing cell was better than that of the CCuF-containing cell.

**Figure 3:**
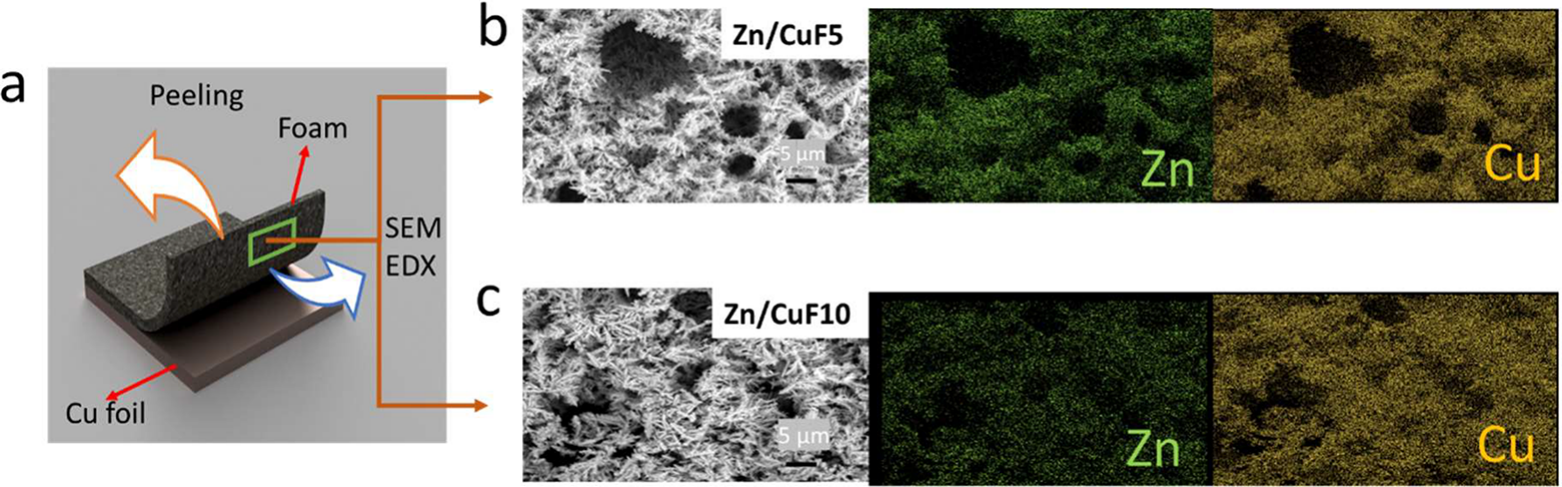
SEM images and EDX mapping images of the bottom of the electrodes after Zn deposition. (a) 3D schematic illustration showing the SEM and EDX results from under the foam for the (b) CuF5 and (c) CuF10 electrodes.

The CuF10 current collector, which was obtained under the same synthesis conditions as CuF5 (except the synthesis time was doubled to 10 s), was almost twice as thick as the CuF5 current collector (Figure S2c). However, it was understood that increasing the electrochemically active surface area was more important than increasing the actual surface area. As seen in the cross-sectional EDX mapping images of the Zn/CuF10 anode electrode obtained after Zn deposition in Figure 1g, it is largely in the parts close to the surface area that were filled with Zn, indicating that the Zn deposition was not able to reach to the depths of the 3D Cu foam structure effectively. To better explain this, the Zn/CuF5 and Zn/CuF10 anode electrodes were peeled off from the copper foil, as illustrated in Figure 3a. Afterward, SEM and EDX elemental mapping analyses were performed on the bottom of these samples, and the results are shown in Figure 3b for Zn/CuF5 and Figure 3c for Zn/CuF10. The obtained EDX data and corresponding ratios are also presented in Figure S10 (panel a for Zn/CuF5 and panel b for Zn/CuF10). The results showed that the Zn/CuF5 anode electrode had approximately 44% Zn content in the bottom layer of the electrode after charging, while this ratio was 28% for the Zn/CuF10 anode electrode. It was thought that the increase in foam thickness prevented Zn deposition from reaching the bottom of the foam to some extent. The reason for this insufficient Zn deposition could be due to the relatively high current density, which impedes the mass transport of Zn ions during deposition. The electrolyte could also be another factor, where much larger pores of Cu have to be deposited to reach the inner depths of the Cu foam. Both of these factors are currently under investigation, where lower current densities and more highly concentrated electrolytes are being utilized to overcome this limitation.

### Electrochemical Performances of Symmetric Cells

**Figure 4:**
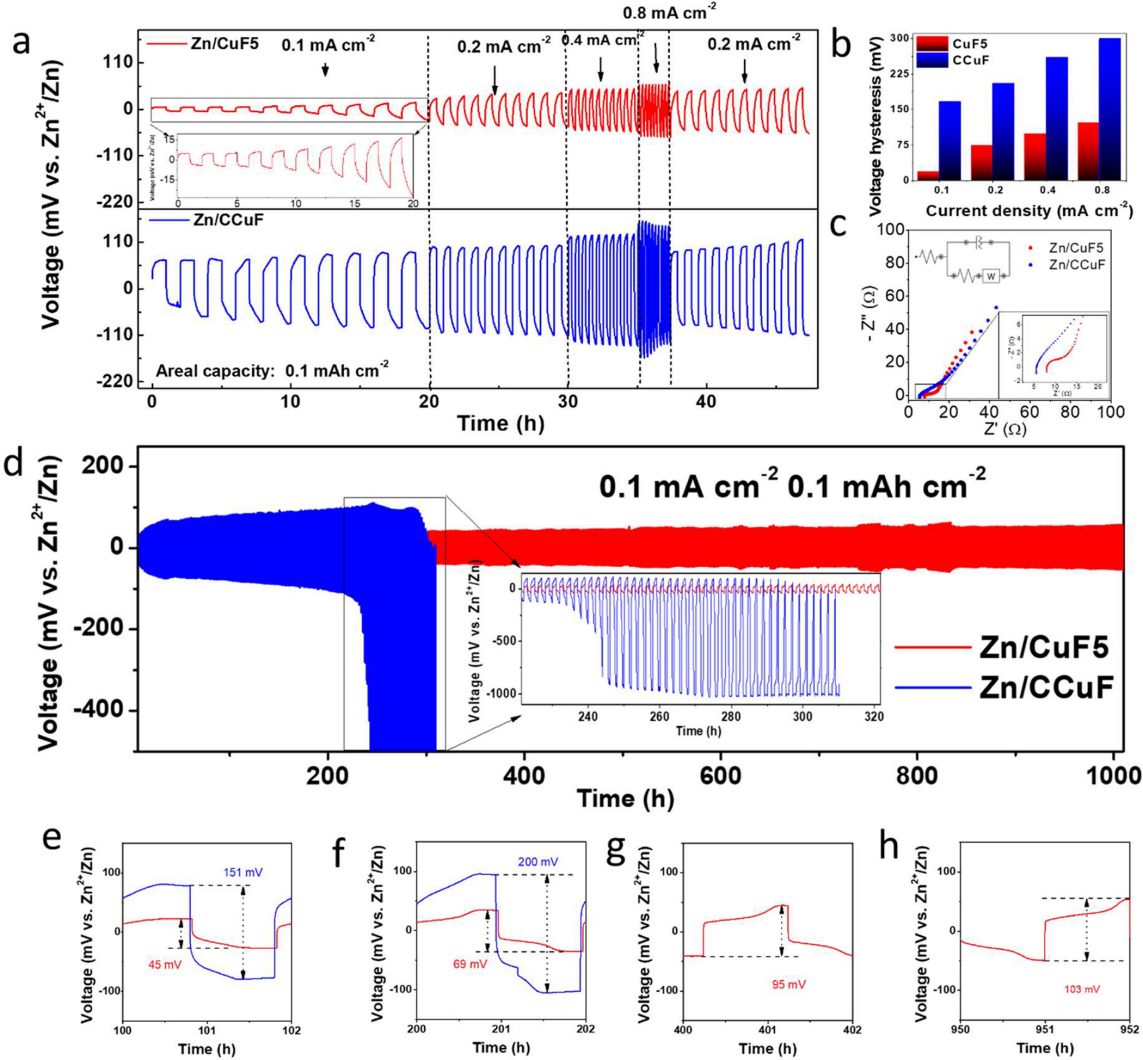
Zn/CuF5 and Zn/CCuF anode electrode performances in symmetric cells. (a) Voltage–time profiles of symmetric cells at different current densities (0.1, 0.2, 0.4, and 0.8 mA cm^–2^; 0.1 mAh cm^–2^ constant areal capacity). (b) Voltage hysteresis bar graph of symmetric cells at different current densities. (c) Nyquist plots of half cells containing CuF5 and CCuF electrodes. (d) Long-term cycling voltage–time profile of symmetric cells at 0.1 mA cm^–2^ current density (0.1 mAh cm^–2^ constant areal capacity). Long-term voltage–time profiles at (e) 100, (f) 200, (g) 400, and (h) 950 h.

The long-term electrochemical cycling stability of the CuF5 electrode was evaluated with a Zn/CuF5//Zn/CuF5 symmetric cell in a 2 M ZnSO_4_ electrolyte. The CCuF electrode was also tested with a Zn/CCuF//Zn/CCuF symmetric cell. Both cells were assembled under the same conditions. The Zn/CuF5//Zn/CuF5 and Zn/CCuF//Zn/CCuF symmetric cells were tested at different current densities (0.1, 0.2, 0.4, and 0.8 mA cm^–2^), limited to an areal capacity of 0.1 mAh cm^–2^. Ten cycles were completed at each current density, followed by a relaxation cycle at a low rate, and the resulting galvanostatic charge/discharge curves are depicted in Figure 4a. From this figure (voltage difference between the charge peak and discharge peak), the voltage hysteresis values were calculated, and the average values with respect to different current densities were plotted, as shown in Figure 4b. In the galvanostatic curves of Figure 4a, the voltage hysteresis values obtained at current densities of 0.1, 0.2, 0.4, and 0.8 mA cm^–2^ were 19, 74, 99, and 122 mV for the Zn/CuF5//Zn/CuF5 symmetric cell, respectively, and 166, 205, 260, and 299 mV for the Zn/CCuF//Zn/CCuF symmetric cell, respectively. The Zn/CuF5//Zn/CuF5 symmetric cell exhibited very low voltage hysteresis at all current densities compared to the Zn/CCuF//Zn/CCuF symmetric cell. This may be due to the fact that the pore size of the CCuF current collector was around 300 μm on average, and the active surface area per unit volume was very low (Figure S11a). This was attributed to the fact that although the CuF5 current collector electrode had a very low thickness (around 38 μm, Figure 1f), the active surface area per unit volume was much higher due to the densely stacked macro/microporous copper structure. The macropores of the CuF5 current collector had an average pore size of 45 μm and were surrounded by walls with a thickness of about 20 μm (Figure S11b). These walls were also composed of micropores with a width of about 1.5 μm (Figure S11c). The Nyquist plots in Figure 4c show dramatic differences for the CuF5 and CCuF containing cells. The equivalent circuit for the fit is also shown as an inset in the figure. The charge transfer resistance corresponding to the high-frequency region was 4.45 Ω for the Zn/CuF5 anode electrode, while for the Zn/CCuF anode electrode, it was calculated to be 16.4 Ω. The Zn/CuF5 anode electrode showed about 4 times lower resistance. The lower charge transfer resistance also further supports what we obtained in the electrochemical performance data. This was generally attributed to the large electrochemically active surface area of the 3D structures, which enhanced the electrode–electrolyte interface interaction.^34^ In addition, symmetric cells with the CuF10 and CuF20 electrodes were prepared under the same conditions. The galvanostatic charge/discharge curves and voltage hysteresis bar graph of the symmetric cells are given in Figure S12. The Zn/CuF10//Zn/CuF10 and Zn/CuF20//Zn/CuF20 symmetric cells exhibited similar characteristics. In cases where the current density could not be distributed homogeneously, there was no uniform Zn deposition on the electrode surface, and shape deformations and passivation were observed over time. High voltage hysteresis and dendritic growth following passivation cause cells to become unusable in a short time.^35^ This claim was proved by the Zn/Cu foil//Zn/Cu foil symmetric cell in Figure S13, where the commercial Cu foil-containing cell experienced a short circuit in a very short time (after about 30 h), whereas the 3D porous-containing symmetric cells (Zn/CuF10//Zn/CuF10 and Zn/CuF20//Zn/CuF20) did not reveal such a feature and cycled well. The foam thickness increased with the deposition time (Figures 1f and S2c). Accordingly, the surface area of the CuF10 and CuF20 current collectors was expected to be larger than that of the CuF5 electrode, and this seemed to contribute positively to the initial voltage hysteresis. However, the large surface area caused side reactions, and polarization also occurred at the electrode; furthermore, very high voltage hysteresis was observed in the next cycles.^36^ When the galvanostatic charge/discharge curves at a current density of 0.1 mA cm^–2^ and an areal capacity of 0.1 mAh cm^–2^ in Figure S13 were examined, they proved the above-mentioned claim. It was seen that the voltage hysteresis of the symmetric cell prepared with the electrode with the largest surface area (CuF20) increased rapidly after about 100 h and increased from 50 to ∼800 mV (at 192 h). While CuF10 continued to be stable until about 350 h, it was seen that the cell was gradually polarized, and a voltage hysteresis of ∼1200 mV was obtained at ∼500 h. In Figure 4d, the long-term cycling stability of the Zn/CuF5//Zn/CuF5 symmetric cell with the Zn/CCuF//Zn/CCuF symmetric cell at a current density of 0.1 mA cm^–2^, limited to a capacity of 0.1 mAh cm^–2^, was investigated. In general, the Zn/CuF5//Zn/CuF5 symmetric cell maintained cycle stability for 1000 h and completed the stability test successfully. However, the Zn/CCuF//Zn/CCuF symmetric cell exhibited a high voltage hysteresis after about 220 h, and the cell became nonfunctional. In addition, the galvanostatic charge/discharge curves of the cells are shown in more detail in Figure 4e–h for further examination. In Figure 4e–h, the Zn/CuF5//Zn/CuF5 symmetric cell showed voltage hysteresis values of 45, 69, 95, and 103 mV at 100, 200, 400, and 950 h, respectively, while the Zn/CCuF//Zn/CCuF symmetric cell showed voltage hysteresis values of 151 and 200 mV at 100 and 200 h (after 220 h, the cell stopped working due to its high polarization, and no voltage hysteresis was observed).

### Electrochemical Performances of Full Cells

The Zn/CuF5 and Zn/CCuF anode electrodes were assembled against the α-MnO_2_ cathode to fabricate Zn/CuF5//α-MnO_2_ and Zn/CCuF//α-MnO_2_ full cell Zn-ion batteries. Here, typical powder-type MnO_2_ nanorods were synthesized by a hydrothermal approach.^20^Figure S14a shows an SEM image of the produced α-MnO_2_ nanorods, and the XRD spectrum in Figure S14b proved that the product was in the α-MnO_2_ phase (JCPDS No: 44-0141).^28^ For a decent comparison, cathode electrodes loaded with identical amounts of active materials (approximately 1.0 mg cm^–2^) were used for all full-cell measurements.

**Figure 5:**
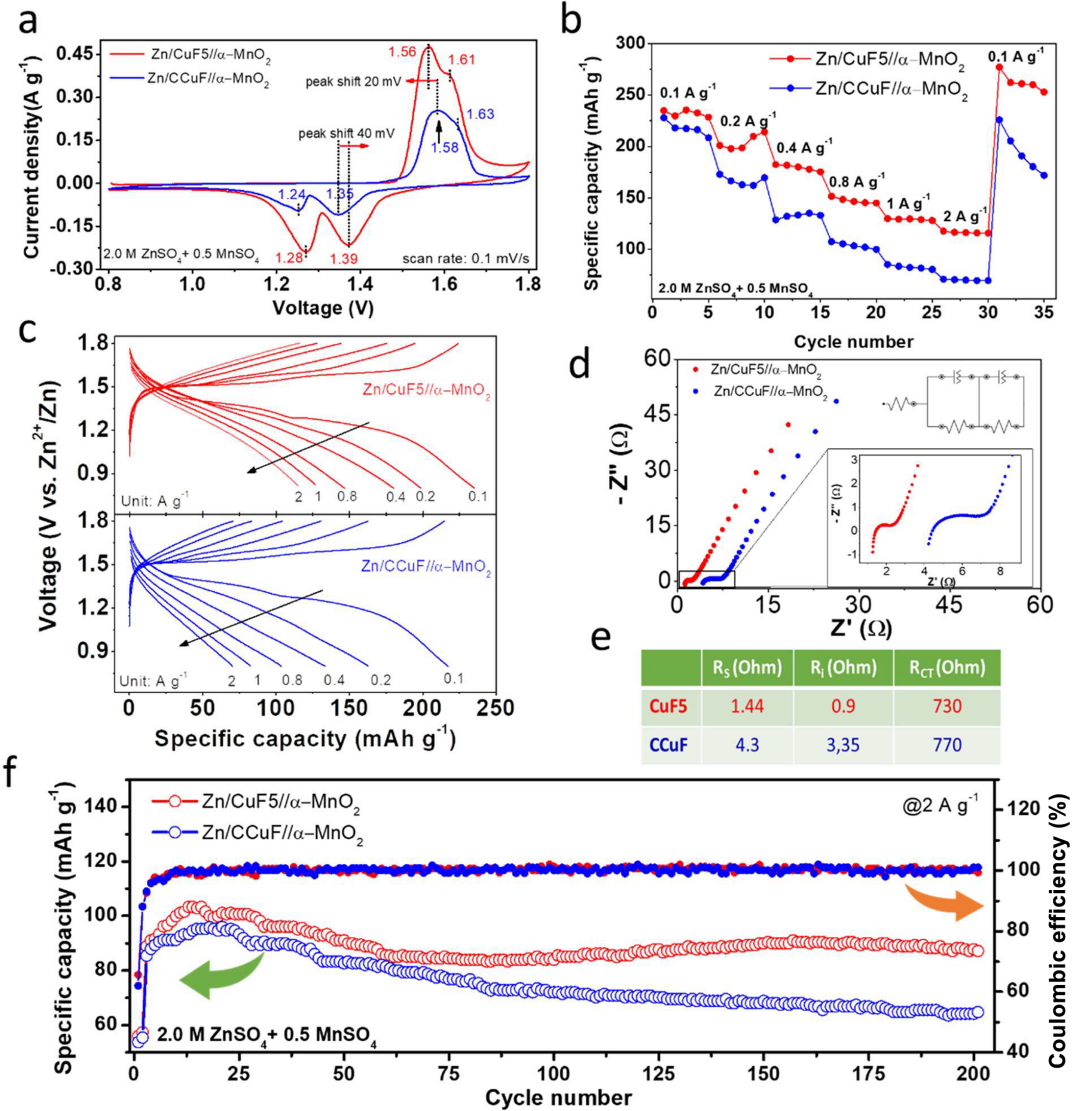
Full-cell performances of Zn/CuF5/α-MnO_2_ and Zn/CCuF/α-MnO_2_ Zn-ion batteries. (a) CV profiles at 0.1 mV s^–1^ within the range of 0.8–1.8 V. (b) Rate performance at current densities from 0.1 to 2.0 A g^–1^. (c) Discharge and charge curves at current densities from 0.1 to 2 A g^–1^. (d) Nyquist plots of the cells and an equivalent circuit model. (e) Table of resistance values obtained after fitting the Nyquist plots to the equivalent circuit model. (f) Long-term cycling data and Coulombic efficiencies at 2 A g^–1^.

The CV curves of the Zn/CuF5//α-MnO_2_ and Zn/CCuF//α-MnO_2_ Zn-ion batteries, which were run between 0.8 and 1.8 V at a scan rate of 0.1 mV s^–1^, are compared in Figure 5a. Two cathodic peaks at 1.39V/1.28 V and two overlapping anodic peaks at 1.56V/1.61 V were observed in the cycles. These two well-separated reversible redox peaks belong to a two-step reaction corresponding to the insertion and extraction of H^+^ (1.39 V/1.61 V) and Zn^2+^ (1.28 V/1.56 V).^37,38^ Furthermore, as shown in Figure 5a, comparing the commercial copper foam anode (Zn/CCuF) with the 3D porous anode (Zn/CuF5), the CuF5-fabricated full cell exhibited a smaller polarization of approximately 60 mV and a higher current density, albeit the same loading, indicating the better electrochemical storage property of the Zn/CuF5 anode electrode.^28,39^ Furthermore, the rate capabilities of the Zn/CuF5//α-MnO_2_ and Zn/CCuF//α-MnO_2_ full cells are shown in Figure 5b, where at current densities of 0.1, 0.2, 0.4, 0.8, 1, and 2 A g^–1^, the Zn/CuF5 anode electrode presented superior discharge capacities of 235, 198, 180, 146, 126, and 115 mAh g^–1^, respectively, while the Zn/CCuF anode electrode achieved capacities of 216, 163, 134, 102, 82, and 70 mAh g^–1^, respectively. After the current density returned to 0.1 A g^–1^, the capacity of the Zn/CuF5 anode electrode increased to 262 mAh g^–1^, while that of the Zn/CCuF anode electrode remained around 190 mAh g^–1^. This indicated that the Zn/CuF5 anode electrode possessed an excellent structural stability. Figure 5c shows the charge and discharge profiles of the full cells operated at different current densities. It can be seen that the cell containing the CuF5 current collector electrode provided a lower charge plateau and a higher discharge plateau than the CCuF electrode (the data obtained at 0.1 and 2 A g^–1^ are also given in Figure S15). The lower voltage gap indicated a lower polarization of the full cell, which was attributed to the improved kinetics of Zn deposition and stripping on the anode side, as other conditions were kept the same.^40^ For the full cells of the ZIB system, EIS was performed. Nyquist plots and an equivalent circuit model for fitting are given in Figure 5d. The resistance values were calculated by fitting the Nyquist plots to the equivalent circuit model and are listed in Figure 5e. The fitting analysis showed that the electrolyte resistance (*R*_s_), interfacial resistance (*R*_i_), and charge transfer resistance (*R*_ct_) for the Zn/CuF5/MnO_2_ full cell were 1.44, 0.9, and 730 Ω, respectively, while the *R*_s_, *R*_i_, and *R*_ct_ values for the Zn/CCuF/MnO_2_ full cell were 4.30, 3.35, and 770 Ω, respectively. The *R*_ct_ value of the Zn/CuF5//MnO_2_ battery was much lower than that of the Zn/CcuF//α-MnO_2_ battery, which indicated a much more effective electron transfer leading to a better electrochemical activity in the Zn/CuF5//α-MnO_2_ full cell.^41^Figure 5f shows the long-term cycling performances of the batteries at a current density of 2 A g^–1^. The capacities of the Zn/CuF5//α-MnO_2_ and Zn/CCuF//α-MnO_2_ batteries increased to 104 and 94 mAh g^–1^ after about the first 15 cycles, respectively. The sudden increase in capacity in the first few cycles is a phenomenon seen in most aqueous Zn-ion batteries utilizing iron oxide as cathodes. It is speculated that there are two main reasons for this behavior. First, it is believed that this phenomenon may be due to inadequate diffusion of the electrolyte into the cathode electrode, and second, it is ascribed to the activation of the MnO_2_ cathode.^42,43^ The subsequent decrease in capacity can be related to the MnO_2_ lattice deformation or irreversible Mn dissolution at the cathodes.^44^ For the Zn/CuF5//α-MnO_2_ battery, the capacity started to increase slightly after about the 75th cycle and stabilized at 90 mAh g^–1^. This could be due to the presence of MnSO_4_ in the electrolyte, which contributed to the amount of MnO_2_ through electrochemical deposition during cycling.^41^ In sharp contrast to the excellent performance of the CuF5-containing full cell, the capacity of the Zn/CCuF//α-MnO_2_ battery dramatically decreased to a capacity of 64 mAh g^–1^ after 200 cycles.

## Conclusion

In summary, Zn/CuF was prepared with a 3D porous copper foam (CuF) current collector fabricated by the DHBT method. The open pore structure of the 3D porous dendritic copper scaffold of the CuF5 current collector with excellent electrical properties that was synthesized in 5 s allowed for a very low zinc nucleation overpotential and uniform deposition/stripping of Zn active material during charging and discharging of the ZIB. During 500 h of Zn deposition/stripping on the CuF5 current collector, a Coulombic efficiency of around 99% (at a 1 mAh cm^–2^ capacity and a current density of 5 mA cm^–2^) was achieved, along with fast electrochemical kinetics and low polarization. Moreover, the symmetric cell exhibited low voltage polarization and a stable voltage hysteresis profile for 1000 h. Furthermore, full cells containing the Zn/CuF anode, α-MnO_2_ nanoneedles, and an aqueous electrolyte containing Zn^2+^ and Mn^2+^ were fabricated. It reached a maximum capacity of 266 mAh g^–1^ at a current density of 0.1 A g^–1^. In this study, we have provided a new route for fabricating very thin, scalable, and inexpensive current collectors with a very high surface area for ZIBs.

## Supporting Information Available

Photographs of the specially manufactured electrode used for foam synthesis, material characterizations of the electrodes (SEM, EDX, and BET data), initial charge curves of the electrodes at different current densities, asymmetric cell tests, symmetric cell tests, Coulombic efficiency data, and SEM image and XRD spectrum of synthesized α-MnO_2_ (PDF)
