# Supplementary material for: Achieving Long Cycle Life of Zn-Ion Batteries through Three-Dimensional Copper Foam
Source: ACS Appl Mater Interfaces. 2024 Apr 25;16(18):23209–19. doi: 10.1021/acsami.4c01028 (PMC11093413; doi:10.1021/acsami.4c01028)
Supplement: Supplementary file 1 — am4c01028_si_001.pdf [file am4c01028_si_001.pdf]

Supporting Information

Achieving Long Cycle Life of Zn-Ion Batteries Through Three-dimensional Copper Foam

Taşkın Çamurcu[1,3], Erhan Demirbaş[1], Mehmet Nurullah Ateş[2,3]**

[1] Gebze Technical University, Department of Chemistry, Gebze, Kocaeli 41420, Türkiye

[2] Bogazici University, Department of Chemistry, Bebek, Istanbul 34342, Türkiye

[3] TÜBİTAK Rail Transport Technologies Institute, Energy Storage Division, TÜBİTAK Gebze Campus, Gebze, Kocaeli 41470, Türkiye

Corresponding authors. E-mail address: mehmet.ates@boun.edu.tr (Mehmet Nurullah Ateş), erhan@gtu.edu.tr (Erhan Demirbaş)

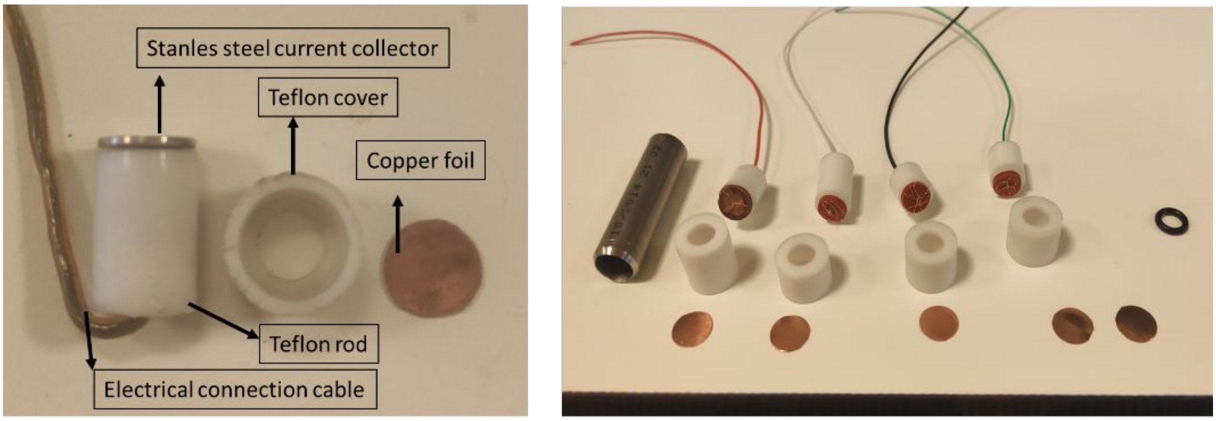

Figure S1. Photos of the special electrode used for the electrochemical synthesis of nano-porous copper foam (CuF).

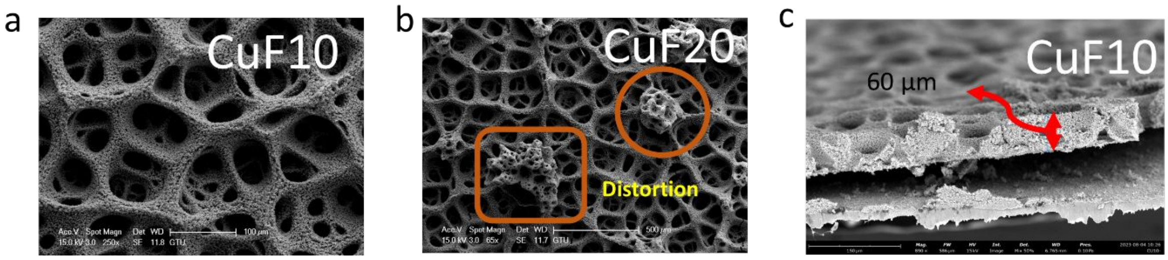

Figure S2. SEM image (a) CuF10, (b) CuF20 and (c) CuF10 cross-section thickness measurements.

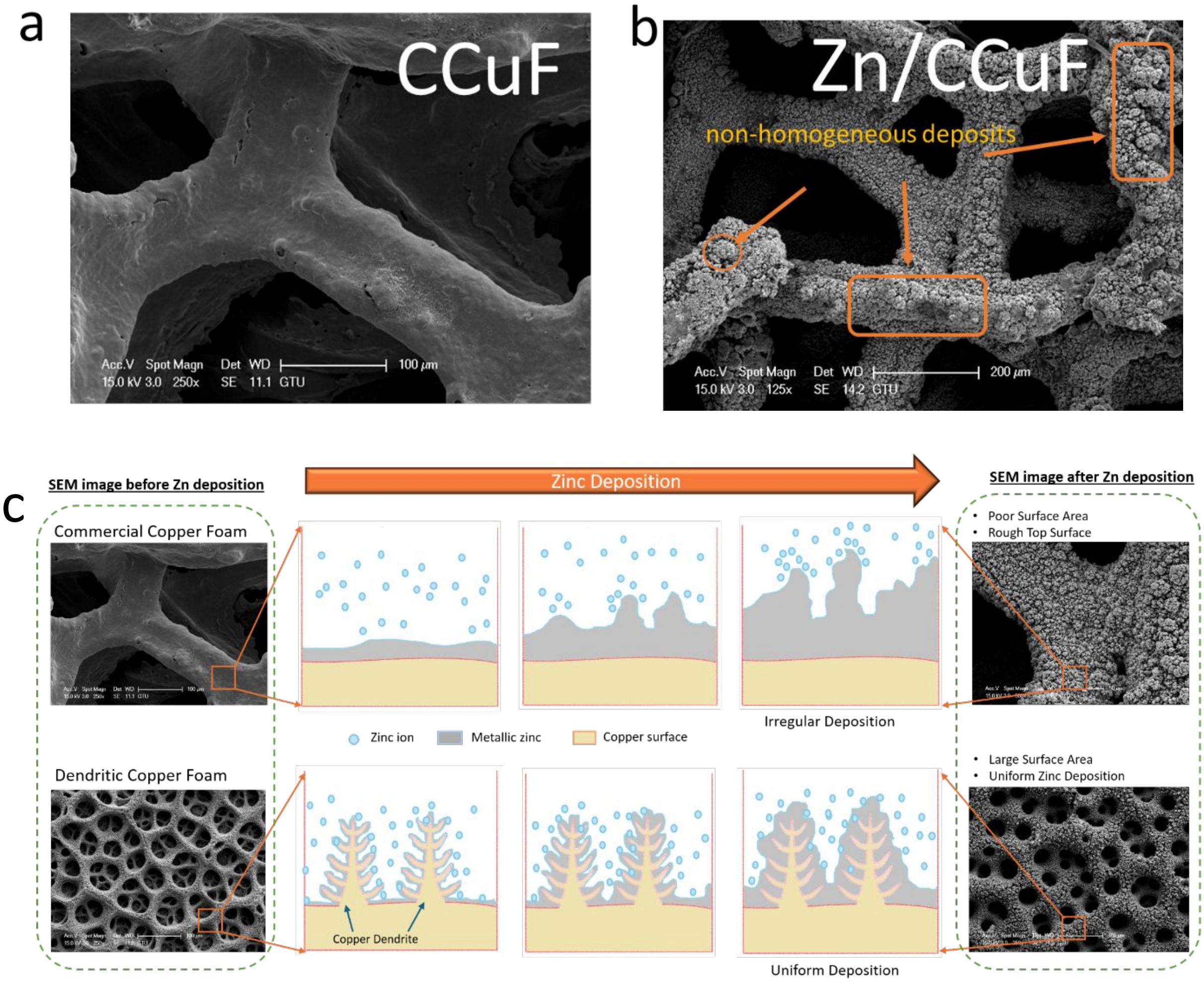

Figure S3. SEM image of (a) commercial copper foam (CCuF), (b) Zn deposited CCuF and (c) a schematic of growth mechanism of Zn in commercial foam versus the foam developed in this study.

a

Element Number	Element Symbol	Element Name	Atomic Conc.	Weight Conc.
29	Cu	Copper	13.020	12.700
30	Zn	Zinc	86.980	87.300

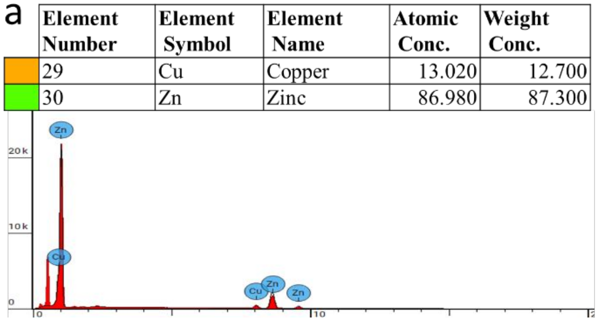

b

Element Number	Element Symbol	Element Name	Atomic Conc.	Weight Conc.
29	Cu	Copper	46.509	45.800
30	Zn	Zinc	53.491	54.200

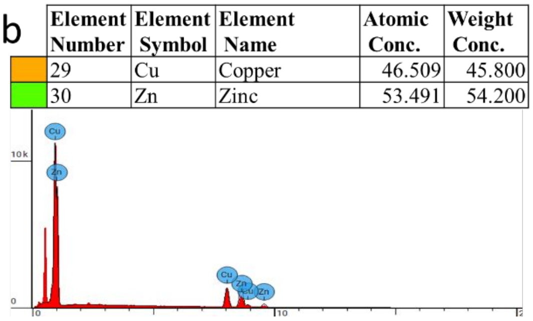

Figure S4. Cross-sectional energy-dispersive X-ray (EDX) spectroscopy elemental mapping by performing a scanning electron microscopy (SEM) (a) percentage of Cu and Zn elements and elemental spectrum for CuF5 and (b) percentages of Cu and Zn elements and elemental spectrum for CuF10.

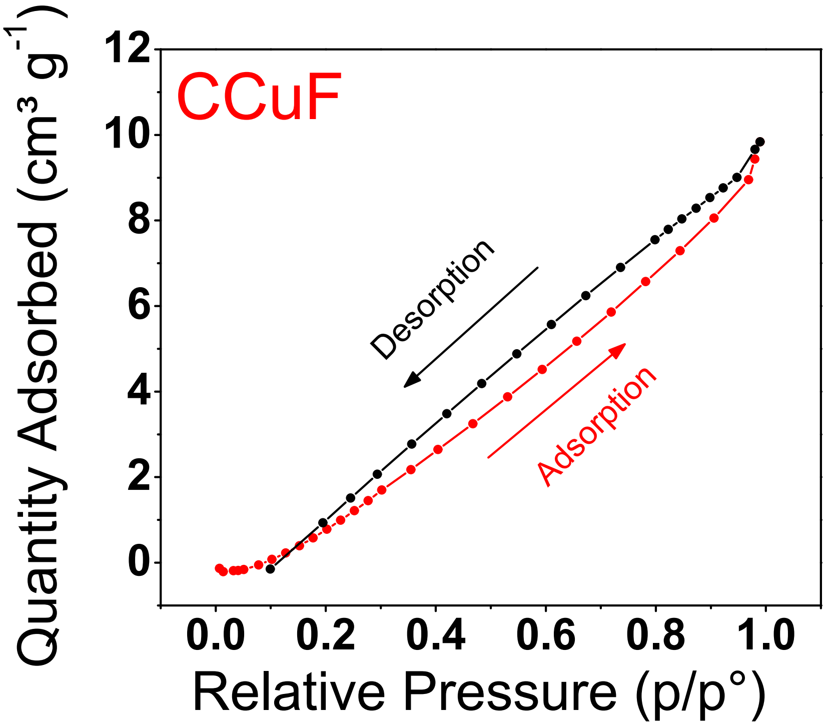

Figure S5. BET analysis of nitrogen adsorption-desorption isotherm for CCuF.

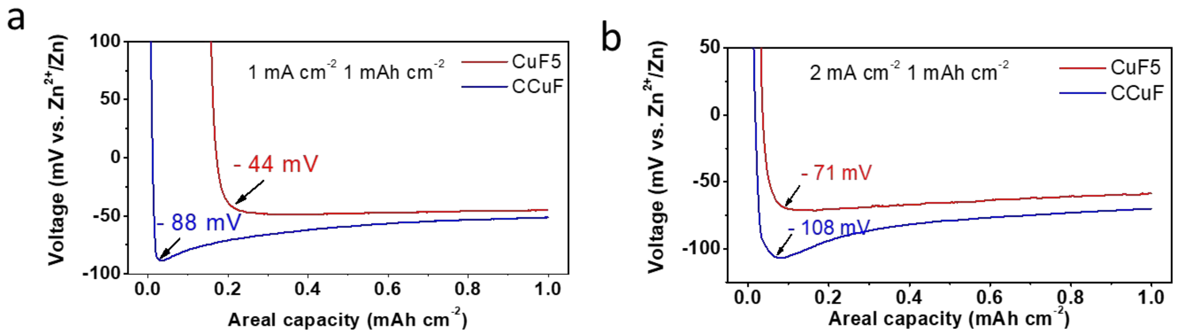

Figure S6. Nucleation overpotentials of CuF5 and CCuF current collectors at an areal capacity of 1 mAh cm^{-2} (a) at 1 mA cm^{-2} and (b) at 2 mA cm^{-2}.

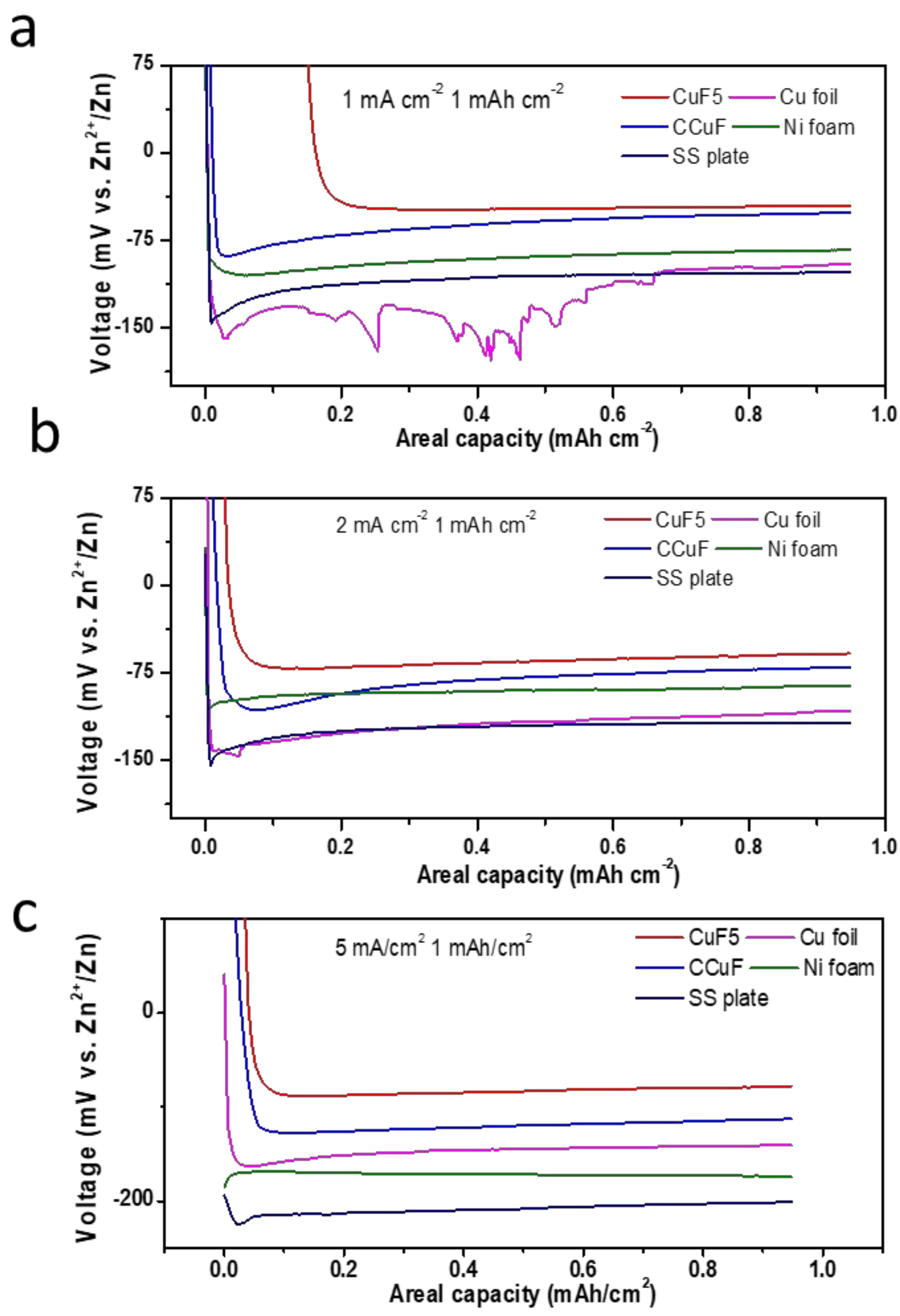

Figure S7. Nucleation overpotentials of Zn deposition on CuF5, CCuF, Cu Foil, Ni Foam and stainless-steel plate (S.S. plate) in asymmetric cells (versus Zn electrode) at constant areal capacity of 1 mAh cm^{-2} (a) 1 mA cm^{-2}, (b) 2 mA cm^{-2} and (c) 5 mA cm^{-2}.

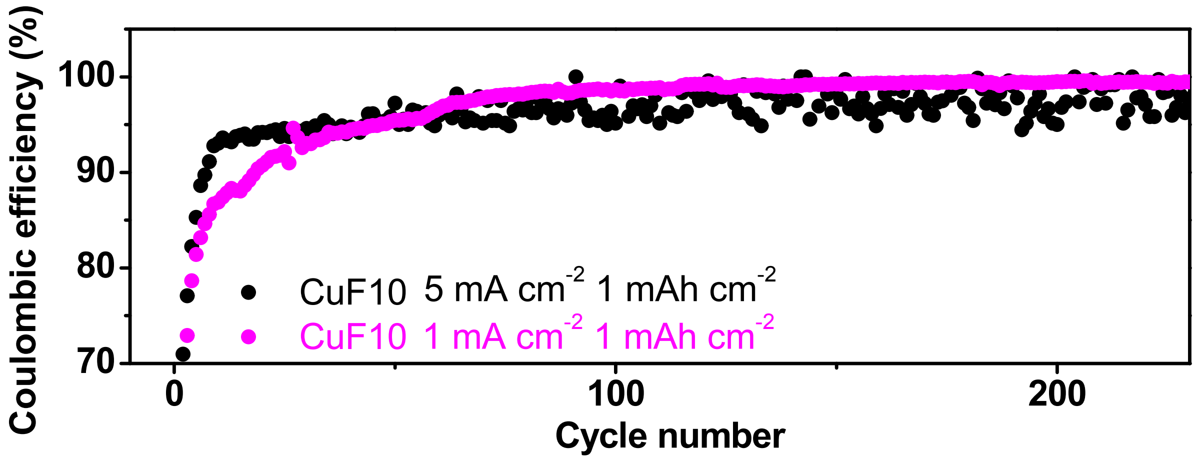

Figure S8. Corresponding columbic efficiencies of long-term cycles of CuF10 current collector in asymmetric cell (versus Zn electrode) at current densities of 1 mA cm^{-2} and 5 mA cm^{-2} (an areal capacity of 1 mAh cm^{-2}).

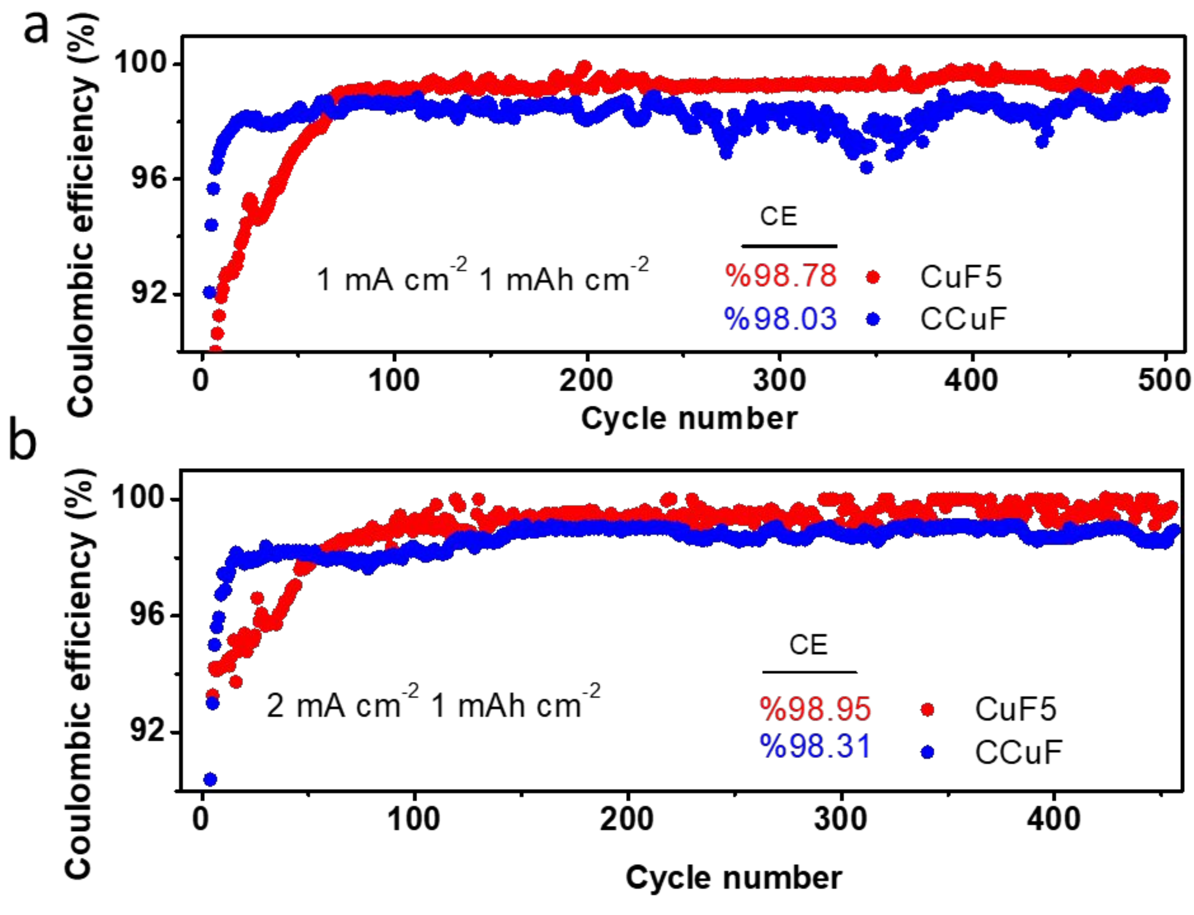

Figure S9. Corresponding columbic efficiencies of long-term cycles of CuF5 and CCuF current collectors at a constant 1 mAh cm^{-2} areal capacity (a) at 1 mA cm^{-2} and (b) at 2 mA cm^{-2}.

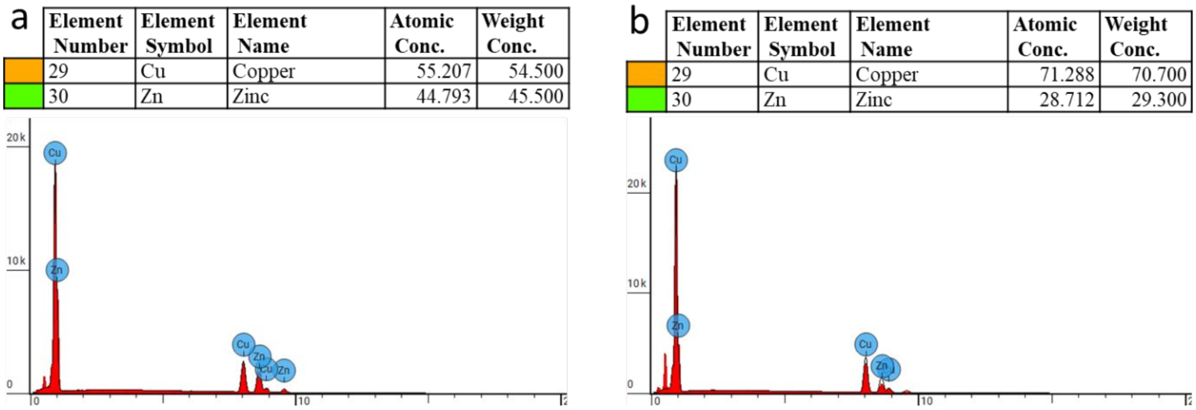

a

Element Number	Element Symbol	Element Name	Atomic Conc.	Weight Conc.
29	Cu	Copper	55.207	54.500
30	Zn	Zinc	44.793	45.500

b

Element Number	Element Symbol	Element Name	Atomic Conc.	Weight Conc.
29	Cu	Copper	71.288	70.700
30	Zn	Zinc	28.712	29.300

Figure S10. Percent elemental content and EDX spectrum of the bottom sections peeled off the copper foil (a) CuF5 and (b) CuF10.

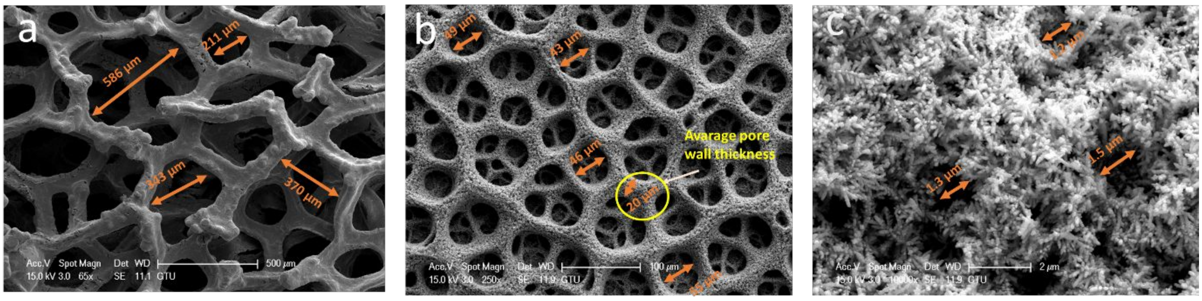

Figure S11. Measurement of pore diameters with the image-j program. (a) CCuF, (b) CuF5 macro pores and (c) CuF5 micro pores.

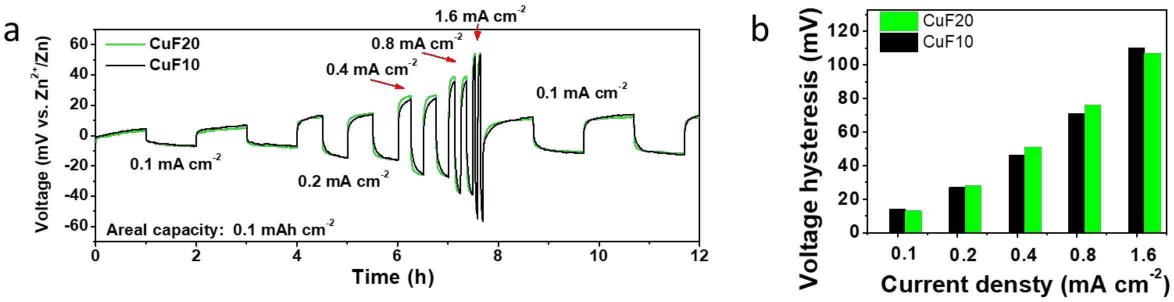

Figure S12. Zn/CuF10 and Zn/CuF20 anode electrodes for symmetrical cells (a) galvanostatic charge/discharge curves at different current densities and (b) voltage hysteresis bar graph.

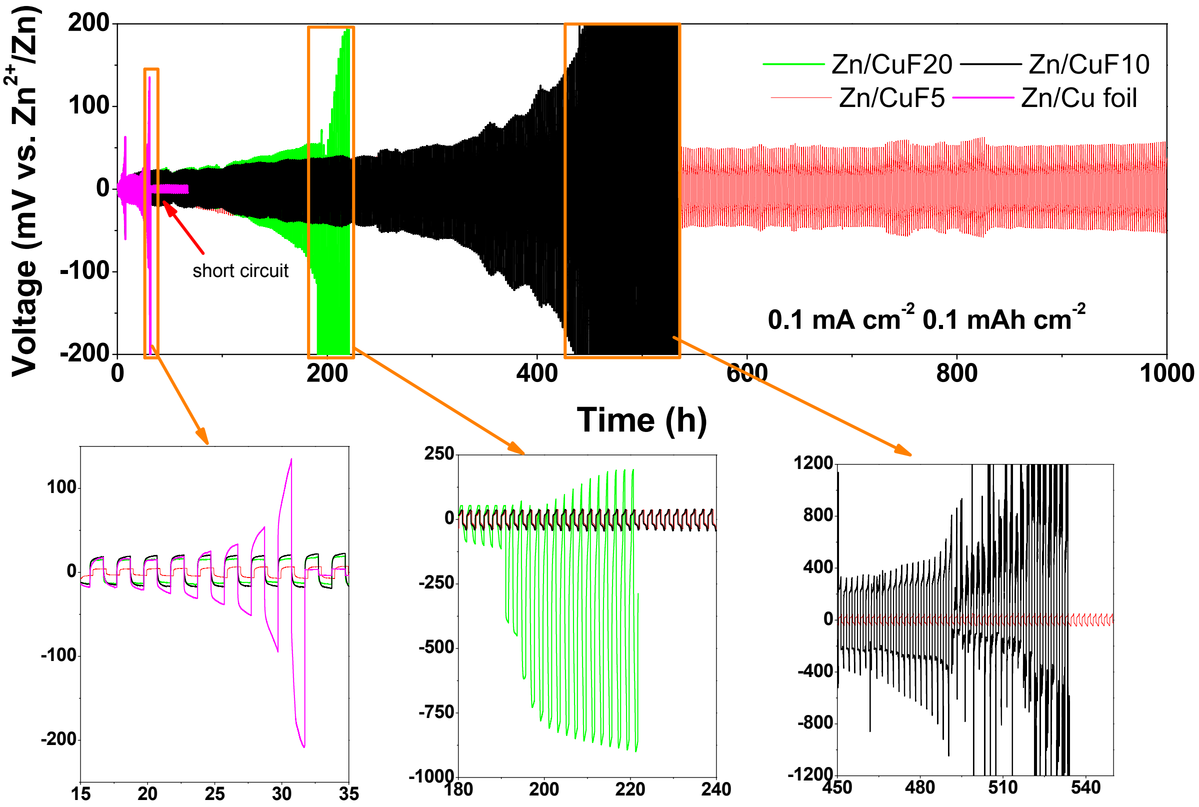

Figure S13. Long term galvanostatic charge/discharge curves of Zn/Cu Foil, Zn/CuF10 and Zn/CuF20 anode electrodes in symmetric battery systems.

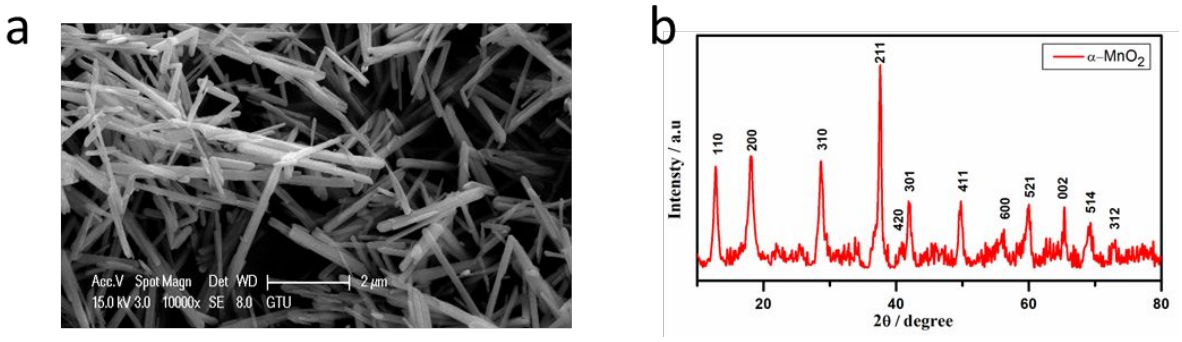

Figure S14. SEM images of (a) α-MnO_2 powder and b) XRD spectra.

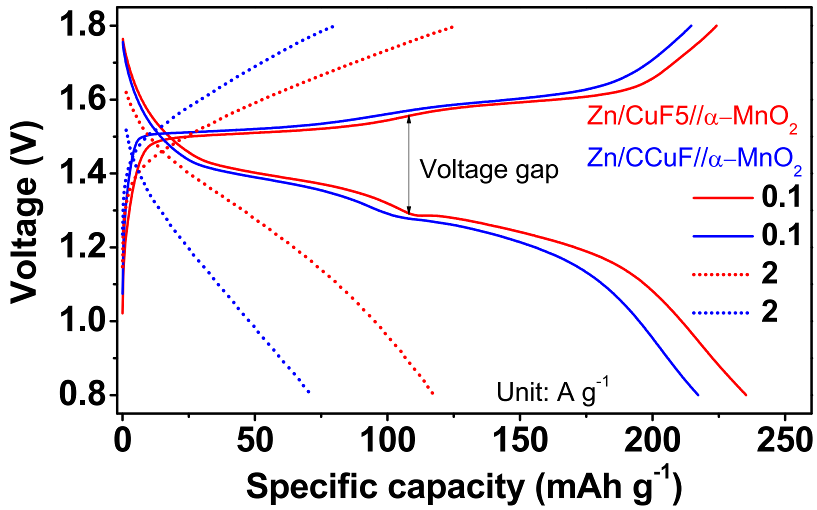

Figure S15. Galvanostatic charge/discharge curves of Zn/CuF5//α-MnO2 and Zn/CCuF//α-MnO2 full cells at 0.1 A g^{-1} and 2 A g^{-1}.
